# Anti-abortion laws—the antithesis of the fundamental rights of women

**DOI:** 10.1016/j.lanepe.2021.100111

**Published:** 2021-04-16

The world celebrated International Women's Day on March 8, 2020, to commemorate "Women in leadership: Achieving an equal future in a COVID-19 world", a theme that was aligned with the priority theme of the UN 65th session of the Commission on the Status of Women: “Women's full and effective participation and decision making in public life, as well as the elimination of violence, for achieving gender equality and the empowerment of all women and girls”. In light of this progressive theme, it is important to highlight one of the regressive laws—the anti-abortion laws—that fundamentally prevent a significant proportion of women from achieving the above goals. Anti-abortion laws inhibit the right of women to freely choose to have an abortion. These laws are regressive, shaped by political conservatism and moral dogmatism, and are the antithesis of the fundamental rights of women.

There are currently 26 countries where all abortions are prohibited (category I), 39 countries where abortion is permitted only when women's life is at risk (category II), and 56 countries where abortion is permitted to preserve women's health (category III). The more permissive categories include 14 countries that allow abortion on social or economic grounds (category IV), and 67 countries that allow abortion on request (category V). Although 59% of women of reproductive age worldwide, representing 970 million women, live in countries where they can exercise their abortion rights (category IV and V), 41%, representing 690 million women, live under restrictive laws. In Europe, abortion is legal in most countries, despite a wide variation in the restrictions under which it is permitted. The exceptions are Malta, Vatican City, San Marino, Liechtenstein, Andorra, and Poland, where abortion is illegal or severely restricted. Malta is the only country in the European Union that bans abortion in all cases (category I).

All of the formerly Communist countries in Europe have liberal abortion laws, with the exception of Poland, which has restricted its abortion laws after decades of permissive legislation during the Polish People's Republic. Abortion in Poland is now allowed only in cases where a woman's life or health is endangered by the continuation of pregnancy or when the pregnancy is a result of rape or incest. In a landmark ruling on Oct 22, 2020, Poland's Constitutional Tribunal, consisting mainly of judges appointed by the ruling party Law and Justice, declared the law authorising abortions for malformed foetuses to be unconstitutional. In 2019, 98% of abortions were carried out on these grounds, which implies that the ruling has banned the vast majority of abortions in the country. The court justified its ruling on the grounds that "an unborn child is a human being" and therefore it deserves protection under Poland's constitution, which ensures the right to life. Poland's conservative government supports the ruling, which took effect on Jan 27, 2021. This decision sparked the country's biggest wave of protests in its post-communist era. The European Union Commissioner for Human Rights, the Council of Europe, and UN independent human rights experts have all condemned this ruling as a violation of human rights and one that goes against Poland's international human rights obligations. However, the European Parliament has no regulatory authority on abortion rights within a member state.

Such regressive anti-abortion laws not only impinge on the fundamental and reproductive rights of women, but also have serious physical and mental health implications for women bearing fetuses with congenital anomalies. For a larger number of serious cases, it should be considered unethical to make women go through a full-term pregnancy against their will knowing that the birth will be followed by the death of the infant. Women with the knowledge of bearing fetuses with serious congenital anomalies, or a child that would die shortly after birth, often present with severe psychiatric disorders. Many children born with severe physical and neurological disabilities are abandoned at birth, and their life expectancy is short. The illegality of abortion leads to unsafe abortion practices, such as the use of a sharp object or wire to break the amniotic sac, self-administering abortifacient, and use of toxic chemicals, which lead to major life-threatening complications resulting from haemorrhage, infection, and injury to the genital tract and internal organs. In countries where abortion is illegal, failed abortions cause around 8–11% of all maternal deaths each year. Legalising abortion will enable the practice of safe abortion procedures and allows for the proper training of medical specialists, which will eventually reduce or even prevent these maternal deaths.

Abortion legislation is largely based on so-called morality policies—policies reflecting high-order moral principles and therefore the positions held by proponents and opponents of restrictive abortion laws are mostly discordant and often irreconcilable. It took 35 years of legal cases, human rights advocacy, feminist activism, and governmental and parliamentary processes to remove one of the most restrictive abortion bans in Ireland in May, 2018. Contesting abortion as a women´s health issue and human rights advocacy was crucial in maintaining political pressure for law reform. Similar strategies and continued pressure from the European Union are needed to reverse Poland's ruling. It is high time to move beyond moral dogma, to protect the fundamental reproductive rights of women.

*The Lancet Regional Health – Europe*

